# Neuroprotective Effects of *Clostridium butyricum* against Vascular Dementia in Mice via Metabolic Butyrate

**DOI:** 10.1155/2015/412946

**Published:** 2015-10-07

**Authors:** Jiaming Liu, Jing Sun, Fangyan Wang, Xichong Yu, Zongxin Ling, Haixiao Li, Huiqing Zhang, Jiangtao Jin, Wenqian Chen, Mengqi Pang, Junjie Yu, Yiwen He, Jiru Xu

**Affiliations:** ^1^Department of Immunology and Pathogenic Biology, School of Medicine, Xi'an Jiaotong University, Xi'an, Shaanxi 710061, China; ^2^School of Environmental Science and Public Health, Wenzhou Medical University, Wenzhou, Zhejiang 325035, China; ^3^Department of Neurology, The Second Affiliated Hospital of Wenzhou Medical University, Wenzhou, Zhejiang 325027, China; ^4^Collaborative Innovation Center for Diagnosis and Treatment of Infectious Diseases, State Key Laboratory for Diagnosis and Treatment of Infectious Diseases, The First Affiliated Hospital, School of Medicine, Zhejiang University, Hangzhou, Zhejiang 310003, China

## Abstract

Probiotics actively participate in neuropsychiatric disorders. However, the role of gut microbiota in brain disorders and vascular dementia (VaD) remains unclear. We used a mouse model of VaD induced by a permanent right unilateral common carotid arteries occlusion (rUCCAO) to investigate the neuroprotective effects and possible underlying mechanisms of* Clostridium butyricum*. Following rUCCAO,* C. butyricum* was intragastrically administered for 6 successive weeks. Cognitive function was estimated. Morphological examination was performed by electron microscopy and hematoxylin-eosin (H&E) staining. The BDNF-PI3K/Akt pathway-related proteins were assessed by western blot and immunohistochemistry. The diversity of gut microbiota and the levels of butyrate in the feces and the brains were determined. The results showed that* C. butyricum* significantly attenuated the cognitive dysfunction and histopathological changes in VaD mice.* C. butyricum* not only increased the levels of BDNF and Bcl-2 and decreased level of Bax but also induced Akt phosphorylation (p-Akt) and ultimately reduced neuronal apoptosis. Moreover,* C. butyricum* could regulate the gut microbiota and restore the butyrate content in the feces and the brains. These results suggest that* C. butyricum* might be effective in the treatment of VaD by regulating the gut-brain axis and that it can be considered a new therapeutic strategy against VaD.

## 1. Introduction

Vascular dementia (VaD), the second most common type of dementia after Alzheimer's disease, results from a reduction in the cerebral blood supply by a blocked or diseased vascular system and leads to a progressive decline in memory and cognitive function [1]. Clinical evidence supports that chronic cerebral hypoperfusion is associated with cognitive decline and hippocampal neuronal injury in neurodegenerative disorders [2, 3]. The possible mechanisms leading to VaD are mainly oxidative stress and apoptosis [4, 5].

An increasing number of studies have investigated the bidirectional signaling of the gut-brain axis [6], which is closely connected between the gut microbiota and brain [7, 8]. Accumulating evidence shows that central nervous system (CNS) disease, such as traumatic brain injury, psychological stress, and Parkinson's disease, could lead to gastrointestinal dysfunction, even causing intestinal barrier dysfunction and compositional changes in gut microbiota. However, little research has determined the effects of normal bacterial colonization on the development and function of the brain. These recent findings on the new role of gut microbiota in the gut-brain axis implicate that gut microbiota could be associated with brain functions as well as neurological diseases via the gut-brain axis. Commensal microbes play critical roles in brain health and disease [9], and dysbiosis may contribute to depression and anxiety [10, 11].* Lactobacillus* and* Bifidobacterium* have been reported to attenuate anxiety, prevent chronic psychological stress [12, 13], reduce apoptosis in several brain regions, and improve learning and memory in mice [14, 15]; these phenomena are often accompanied by changes in the microbial composition and active microbial metabolites in the gut [16, 17]. Overall, the colonization of probiotics may initiate signaling mechanisms affecting neuronal action. Therefore, we hypothesized that commensal intestinal microbiotas communicate with the brain under normal conditions and their presence influences VaD development.

Probiotics are live bacteria that usually colonize the gastrointestinal tract and exert beneficial effects through the modulation of gut microbiota [18]. Probiotics are known to offer protection against various chronic diseases such as constipation, antimicrobial-associated diarrhea, and allergies [19]. Probiotics have been widely accepted as a safe and economical therapeutic option [20]. However, the use of probiotics as treatment protocols is limited, primarily due to a lack of effective data and defined mechanisms of action [21].* Clostridium butyricum* resides in the intestine of healthy animals and humans and is a probiotic that has been characterized for its beneficial effects in gastrointestinal disease both* in vitro* and* in vivo* [22].* C. butyricum* treatment may exert its positive effects via modulating gut microbiota and their metabolic short chain fatty acids (SCFAs). SCFAs, including acetate, propionate, and butyrate, are an important part of these metabolites; they have a crucial role as an energy source for intestinal epithelial cells and have effects on anti-inflammatory properties [23]. Furthermore, butyrate is not restricted to the intestinal tract but can be disseminated systemically and is detected in the brain [24]. Butyrate in the brain can exert neuroprotective effects on neurodegenerative disorders and improve behavioral deficits via the inhibition of histone deacetylases (HDACs) [25].

In this study, we investigated the potential neuroprotective effects and possible mechanisms of* C. butyricum* in mice with VaD. Moreover, we demonstrated that the administration of* C. butyricum* led to a modulation of gut microbiota composition and a change in SCFAs, namely, butyrate. The beneficial effects of* C. butyricum* were associated with a change in butyrate levels in the brain. These results support the notion that the probiotic-gut microbiota-butyrate-brain axis promotes metabolic neuroprotective effects against VaD.

## 2. Materials and Methods

### 2.1. Animals

Male ICR mice (20–25 g, 6 weeks old) were purchased from the Experimental Animal Center of Wenzhou Medical University and maintained under specific pathogen-free (SPF) conditions. Mice were housed in groups (3-4) in plastic cages (27 × 17 × 13 cm, *L* × *W* × *H*) with ad libitum access to food and water under controlled laboratory conditions of temperature (22 ± 2°C) and humidity (55 ± 5%) with a 12 h light/dark cycle (lights on at 08:00 am). The mice were allowed to acclimatize to the laboratory for 1 week prior to beginning of the study. Animals were housed in a specific room, and the treatment groups were separated from each other to avoid cross contamination. All experiments were performed according to animal use guidelines and approved by the Animal Experimentation Ethics Committee of Wenzhou Medical University.

### 2.2. Bacterial Preparation

*C. butyricum* WZMC1016 (CGMCC 9831) was provided by the China General Microbiological Culture Collection Center and was cultured in MRS broth (Hopebio, Qingdao, China) for 24 h in an anaerobic chamber (5% CO_2_) at 37°C.* C. butyricum* was harvested from the MRS broth (4,500 rpm; 15 min) and resuspended in sterile saline. We established the standard curve between the absorbance and colony forming units (CFU) with a positive linear relationship, giving calculated bacteria counts of 5.0 × 10^9^ CFU/mL at an absorbance of 0.25 at 600 nm. The experimental final concentrations were 5.0 × 10^6^ CFU/mL, 5.0 × 10^7^ CFU/mL, and 5.0 × 10^8^ CFU/mL.

### 2.3. Vascular Dementia Mouse Model

Mice were subjected to permanent right unilateral common carotid arteries occlusion (rUCCAO) as a VaD mouse model, as previously described [26, 27]. Mice were anesthetized by an intraperitoneal injection of 400 mg/kg body weight chloral hydrate (Sinopharm Chemical Reagent Co., Ltd., Shanghai, China) and fixed on an operating table. The right common carotid artery was isolated and sutured permanently with a small-diameter silk thread. The rectal temperature was maintained at 37 ± 0.5°C with a homoeothermic pad controlled during the VaD surgical procedure. After skin closure, the mice were returned to their cages and normal feeding was resumed. A 30% drop in the regional cerebral blood flow was considered as a successful VaD mouse model [28, 29]. The sham group underwent the same surgical procedure without carotid ligation.

### 2.4. Experimental Design

Mice were randomly divided into five groups: (1) sham control group (*n* = 12), which was subjected to a sham operation; (2) VaD model group (*n* = 12), which was subjected to rUCCAO; and (3–5)* C. butyricum* groups of fifteen mice each, which were subjected to rUCCAO and treated with a suspension of* C. butyricum* (1 × 10^6^ CFU, 1 × 10^7^ CFU, and 1 × 10^8^ CFU, resp.) that was freshly prepared as previously described. The sham control group and VaD model groups were treated with physiological saline. All animals were treated intragastrically with physiological saline or* C. butyricum* following rUCCAO at doses of 200 *μ*L through a stainless steel gavage needle once daily for 6 weeks (the initial treatment was 24 h after the rUCCAO operation). At the sixth week, behavioral tests were run in the order of the open field test followed by the Morris water maze. During the behavioral evaluations,* C. butyricum* was administered after the tests every day. At the end of the experimental period, the mice were euthanized and tissue samples (colon contents and brain tissue) were collected for analysis.

### 2.5. Open Field Test

Locomotors activity of mice was recorded by digital behavioral system-locomotors module (Digbehav-LA, ShangHai Ji-Liang Software Technology Co., Ltd., China). Chambers (40 × 40 × 60 cm) are connected to computer system by video camera. Chambers were cleaned by acetic acid and 75% alcohol 1 h before the test. The room temperature was kept at 22 ± 2°C and the humidity was 50 ± 5%. All tests were conducted between 9 and 12 o'clock. The mouse was gently placed in the center of the chamber and allowed to move freely. The locomotors activity was recorded by video tracking system for 60 min without any disturbance. After test, the videos were analyzed by the Open Field software of Digbehav-LA system. The total distance travelled and the total time of rest and active in center were recorded for 25–30 min.

### 2.6. Morris Water Maze

The Morris water maze (DigBehv-MG, Shanghai Jiliang Software Technology Co., Ltd., China) consisted of a video capture system, a data analysis system, and a circular plastic water tank (Φ = 120 cm). The tank was filled with water at a depth of 30 cm and divided into four equal hypothetical quadrants. The position of the marks remained unchanged throughout testing. A platform (Φ = 9 cm) was placed into the water at a constant position and submerged 1 cm below the surface of the water. The Morris water maze experiment assesses positional navigation and spatial exploration. In training experiment, on days 1–5, mice were placed in the water at 4 distinct starting quadrant points and trained to find the submerged platform within 60 s. The time spent finding the platform is termed as the escape latency. On the sixth day, the platform was removed from the tank and each mouse was tested in a probe trial in which the time spent in the former platform-containing quadrant was recorded. The Morris water maze testing procedure has been previously described [30].

### 2.7. Fecal Sample Collection and Total DNA Extraction

After the behavior tests, the mice were euthanized and the cecum contents were quickly removed. Fresh samples were collected separately into sterile centrifuge tubes and immediately transferred to the laboratory on ice and stored at −80°C within 15 min of preparation for further analysis. Then, the bacterial genomic DNA was extracted from the fecal samples using the QIAamp DNA Stool Mini Kit (QIAGEN, Hilden, Germany) with the following modifications: the samples were agitated with 100 mg glass beads (0.1 mm) in a mini-bead beater (FastPrep, Thermo Electron Corporation) for 2 min and incubated at 56°C for 1 h in lysis solution containing proteinase K. Finally, the DNA was eluted in 20 *μ*L elution buffer and stored at −20°C for further analysis [31].

### 2.8. PCR-DGGE Analysis

For amplification of the bacterial DNA, the universal bacterial primers 341F and 534R for the V3 regions of the 16S rRNA genes were used with the reaction conditions described by Ling et al. DGGE of the PCR products was performed as described by Muyzer et al. [32] and Ling et al. with a 35 to 50% gradient for intestinal microbiota and a 25 to 55% gradient for ectocervical microbiota, using a D-Code system (Bio-Rad). The sequence analysis of the excised DGGE bands was performed as previously described [31].

### 2.9. Ultrastructure Analysis

After the Morris water maze, the mice were euthanized under anesthesia, and the brains were collected and fixed in 25% glutaraldehyde for 2 h at 4°C, rinsed with PBS, and soaked in osmium tetroxide. Ultrathin sections were prepared and placed onto colloid coated copper grids and double-stained with 0.4% uranyl acetate and 2% lead acetate. Following this process, the ultrastructure of the neurons was observed and photographed using transmission electron microscopy [33].

### 2.10. Histology Analysis

The hippocampal tissues were removed and embedded in paraffin. Prepared sections (5 *μ*m) were stained with either hematoxylin and eosin (HE) or terminal deoxynucleotidyl transferase dUTP nick-end labeling (TUNEL) reagents (TUNEL, Roche Diagnostics, Germany) using standardized protocols and then analyzed and examined by light microscopy. The positive cells were defined as brown cells with apoptotic nuclear features.

### 2.11. Western Blot Analysis

Brain samples were rapidly dissected, rinsed in 1x PBS, and homogenized in an ice-cold homogenization buffer (Beyotime Institute of Biotechnology, Shanghai, China). The samples were then analyzed by Western blot. The proteins were collected and centrifuged at 12,000 ×g for 15 min at 4°C. A BCA protein assay kit (Beyotime Institute of Biotechnology, Shanghai, China) was used to determine the protein concentration. The samples (20 *μ*g) were separated using 12% sodium dodecyl sulfate-polyacrylamide gel electrophoresis (SDS-PAGE) and transferred onto a nitrocellulose membrane (Bio-Rad, Hercules, CA). The membranes were incubated in blocking solution comprising 5% nonfat milk in TBST at room temperature for 1.5 h and then incubated with the primary antibodies diluted in blocking solution individually. The primary antibodies were anti-BDNF, anti-p-Akt, anti-Akt, anti-Bcl-2, and anti-Bax antibody (1 : 1000, Bioworld, USA). After incubation with primary antibodies, the membranes were incubated with HRP conjugated secondary antibodies (1 : 2000, Beyotime Institute of Biotechnology, Shanghai, China) for 1 h at room temperature. The bound antibodies were detected using a chemiluminescence system (ECL Plus, Thermo Scientific, and Rockford, IL). Images were scanned, and the results were quantified using the National Institutes of Health ImageJ software (Bio-Rad Laboratories, Hercules, CA, USA). *β*-actin was used as a loading control.

### 2.12. Immunohistochemistry

The hippocampal tissues were fixed, embedded in paraffin, and sectioned at 5 *μ*m. Immunohistochemistry for BDNF, p-Akt, Bcl-2, and Bax was then carried out on the sections. Slices were incubated with the primary antibodies anti-BDNF, anti-p-Akt, anti-Bcl-2, or anti-Bax (1 : 200, Bioworld, USA) overnight at 4°C. The sections were then incubated with secondary antibodies and visualized using diaminobenzidine (DAB) as the chromagen. Cells with brown granules were considered immunoreactive positive cells. The slides were observed under a microscope and photographed.

### 2.13. Butyrate Assay

The cecocolic contents and brains were quickly removed and accurately weighed. The content of butyrate was measured using an ion chromatograph. An ion chromatograph model (Dionex ICS-1100, Dionex, USA) integrated with a Dionex IonPacAS14 chromatographic column (4 × 250 mm) was used for the chromatographic separation of the butyrate. The mobile phase consisting of the eluent bottle was delivered to a RFIC-ER to produce an ion strength gradient that started at 0.1 mmol/L NaOH with a flow rate of 1.0 mL/min. The column was injected with 50 *μ*L supernatant of brain tissue. The typical retention time for the butyrate was 30 min.

### 2.14. Statistical Analysis

Data of escape latency in the Morris water maze experiment were analyzed by two-way analysis of variance (treatment × trial day, ANOVA) with repeated measures followed by the Bonferroni test. All other data (i.e., open field test, probe trial, Western blot analysis, and butyrate assay) were analyzed with one-way ANOVA followed by Tukey's test. The results are expressed as mean ± standard deviation (SD) or mean ± standard error of the mean (SEM). Behavioral data (i.e., Morris water maze experiment and open field test) are presented as the mean ± SEM. Other data (i.e., Western blot analysis, butyrate assay) are presented as the mean ± SD. Statistical analyses were performed using SPSS statistical software, version 17.0 (SPSS, Chicago, IL, USA). *P* < 0.05 was considered significant.

## 3. Results

### 3.1. *C. butyricum* Improves Spatial Learning Ability

**Figure 1:**
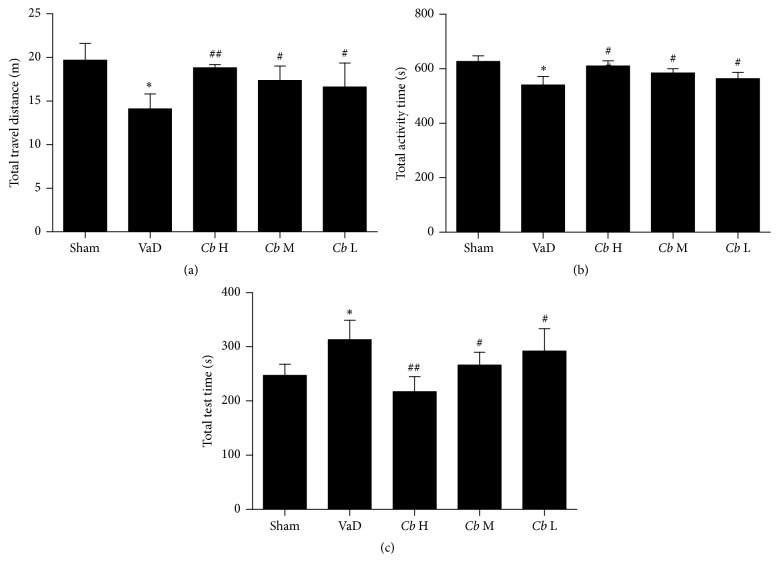
Open field test. (a) The total distance travelled over 1 h, (b) the time spent in the central area, and (c) the total rest time are presented for each group. Sham, sham-operated group; VaD, VaD model group; *Cb* H,* C. butyricum- *(1 × 10^8^ CFU-) treated group; *Cb* M,* C. butyricum-* (1 × 10^7^ CFU-) treated group; and *Cb* L,* C. butyricum-* (1 × 10^6^ CFU-) treated group. Error bars indicate SEM; ^*∗*^ *P* < 0.05 versus sham group; ^#^ *P* < 0.05 and ^##^ *P* < 0.01 versus VaD group.

To verify the effects of sport ability on cognitive improvement, we tested locomotors activity. The total distance travelled and activity time of the VaD group were significantly decreased compared with the sham group (*P* < 0.05), whereas the* C. butyricum*-treated mice showed a significant increase in these measures in comparison with the VaD group (*P* < 0.05, Figures 1(a) and 1(b)). Furthermore, while the rest time of the mice with VaD was significantly increased compared with the sham group (*P* < 0.05, Figure 1(c)), the rest time of the* C. butyricum*-treated mice was dramatically decreased in comparison with the VaD mice (1 × 10^6^ and 1 × 10^7^ CFU: *P* < 0.05; 1 × 10^8^ CFU: *P* < 0.01, Figure 1(c)). The locomotors activity was reduced in the VaD mice. All total distance and total rest and active time in center were altered by* C. butyricum* significantly, indicating a neuroprotective effect in mice model of VaD with concomitant improvement of motor functions.

**Figure 2:**
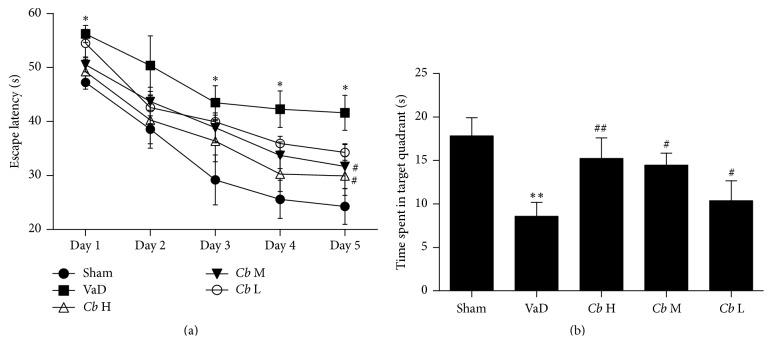
Morris water maze. (a) Escape latency (s). (b) The time spent in the target quadrant (s). Sham, sham-operated group; VaD, VaD model group; *Cb* H,* C. butyricum-* (1 × 10^8^ CFU-) treated group; *Cb* M,* C. butyricum-* (1 × 10^7^ CFU-) treated group; and *Cb* L,* C. butyricum-* (1 × 10^6^ CFU-) treated group. Error bars indicate SEM; ^*∗*^ *P* < 0.05 and ^*∗∗*^ *P* < 0.01 versus sham group; ^#^ *P* < 0.05 and ^##^ *P* < 0.01 versus VaD group.

The Morris water maze is conventionally used to measure cognitive function. In the training trials, all of the groups gradually learned to find the hidden platform and gently shortened their escape latencies. A two-way ANOVA analysis showed that the escape latency in the VaD group was significantly longer than in the sham group on days 1, 3, 4, and 5 (*P* < 0.05, Figure 2(a)), suggesting an impairment in spatial learning of these vascular dementia mice. After treatment with* C. butyricum*, the escape latency on day 5 was decreased significantly as compared to the VaD mice (1 × 10^7^ and 1 × 10^8^ CFU: *P* < 0.05, Figure 2(a)). In the probe trails, the data showed that there was obvious reduction of the time spent in the target quadrant in the VaD group compared to the sham group (*P* < 0.01, Figure 2(b)). The time spent in the target quadrant for the* C. butyricum* treatment group was much longer than in the VaD group (1 × 10^6^ and 1 × 10^7^ CFU: *P* < 0.05; 1 × 10^8^ CFU: *P* < 0.01, Figure 2(b)), indicating that* C. butyricum* treatment could attenuate cognitive impairment of VaD mice.

### 3.2. *C. butyricum* Ameliorated the Morphological Changes in the Hippocampus

**Figure 3:**
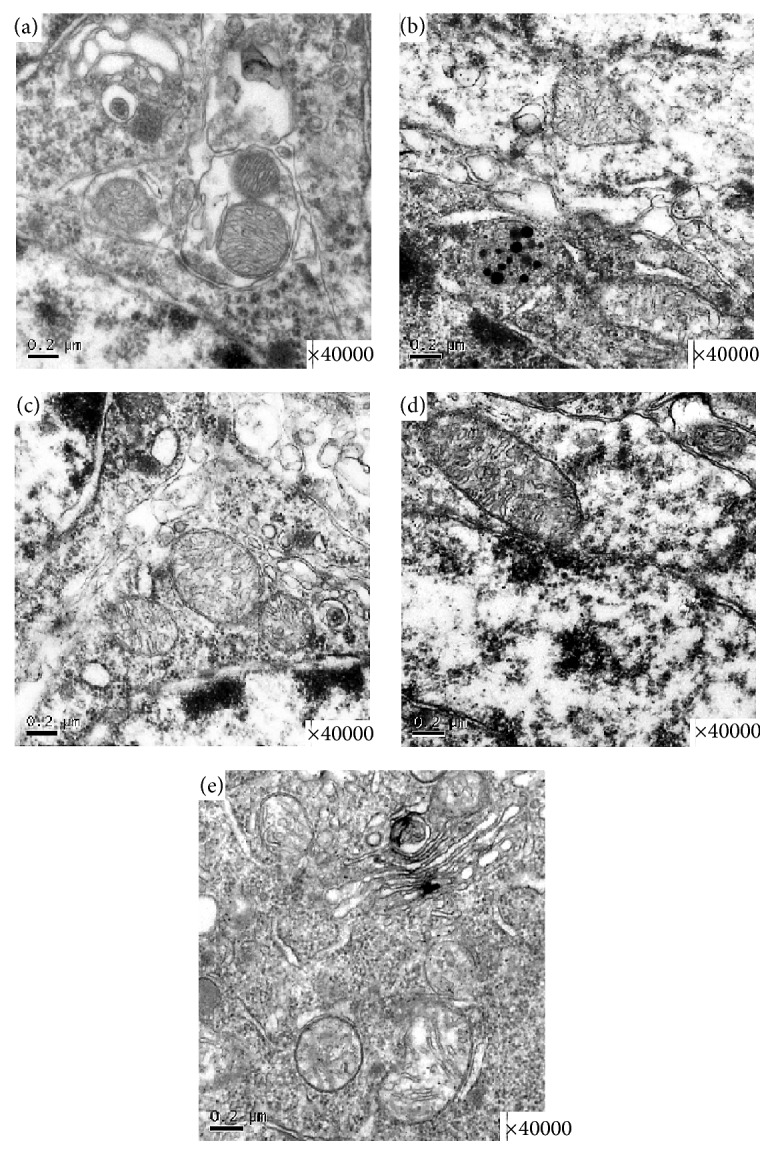
Representative photomicrographs of the ultrastructural changes observed in brain tissue. (a) A representative nucleolus in a hippocampal neuron of the sham-operated group. (b) A representative nucleolus of cerebral ischemia in the VaD group. (c) A representative nucleolus in the *Cb* L group, *Cb* M group (d), and *Cb* H group (e). Sham, sham-operated group; VaD, VaD model group; *Cb* H,* C. butyricum-* (1 × 10^8^ CFU-) treated group; *Cb* M,* C. butyricum-* (1 × 10^7^ CFU-) treated group; and *Cb* L,* C. butyricum-* (1 × 10^6^ CFU-) treated group.

As illustrated by electron microscopy in Figure 3, no destructive changes were observed in the sham group; the neurons contained large oval nuclei with homogeneously distributed euchromatin and clear mitochondria (Figure 3(a)). Irregularly shaped nuclei were observed for neurons in the mice with VaD, along with the appearance of uneven chromatin and swollen mitochondria (Figure 3(b)). By contrast,* C. butyricum* treatment attenuated this impairment in the neuronal ultrastructure in the VaD group (1 × 10^6^ CFU, 1 × 10^7^ CFU, and 1 × 10^8^ CFU: Figures 3(c), 3(d), and 3(e)).

**Figure 4:**
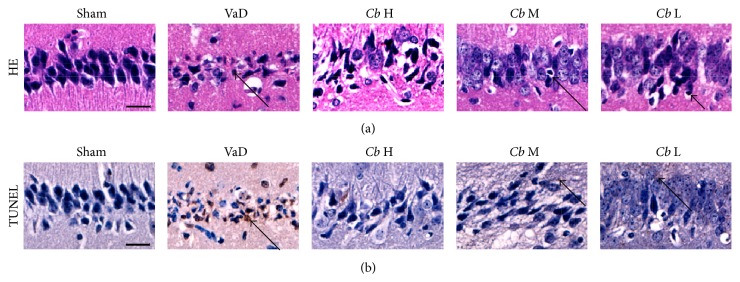
Representative photomicrographs of the histopathological changes in area CA1 of the hippocampus in mice. (a) HE staining. (b) TUNEL staining. Cells with a brown-stained cytoplasm are considered positive. Sham, sham-operated group; VaD, VaD model group; *Cb* H,* C. butyricum-* (1 × 10^8^ CFU-) treated group; *Cb* M,* C. butyricum-* (1 × 10^7^ CFU-) treated group; and *Cb* L,* C. butyricum-* (1 × 10^6^ CFU-) treated group. Magnification: 400x. Scale bar = 20 *μ*m.

As shown in Figure 4, by HE staining, the pyramidal neurons in the CA1 region of the hippocampus lined up in order with round or oval nuclei and clear nucleolus in the sham group. By contrast, the mice subjected to VaD exhibited many morphological changes in the hippocampal CA1 region such as a disappearance of the nucleolus and shrunken neurons due to condensation of the cytoplasm and karyoplasms.* C. butyricum* treatment attenuated the morphological changes of the hippocampal granule cells of VaD mice. By TUNEL staining, the neurons in area CA1 of the hippocampus of mice with VaD showed obvious apoptosis, and the occurrence of apoptosis was significantly ameliorated by the* C. butyricum* treatment; apoptotic features were not observed locally in the* C. butyricum-* (1 × 10^8^ CFU-) treated mice.

### 3.3. *C. butyricum* Treatment Activated BDNF-PI3K/Akt Pathway-Related Proteins (BDNF, p-Akt, Akt, Bcl-2, and Bax) in Mice with VaD as Assessed by Western Blot and Immunohistochemistry

**Figure 5:**
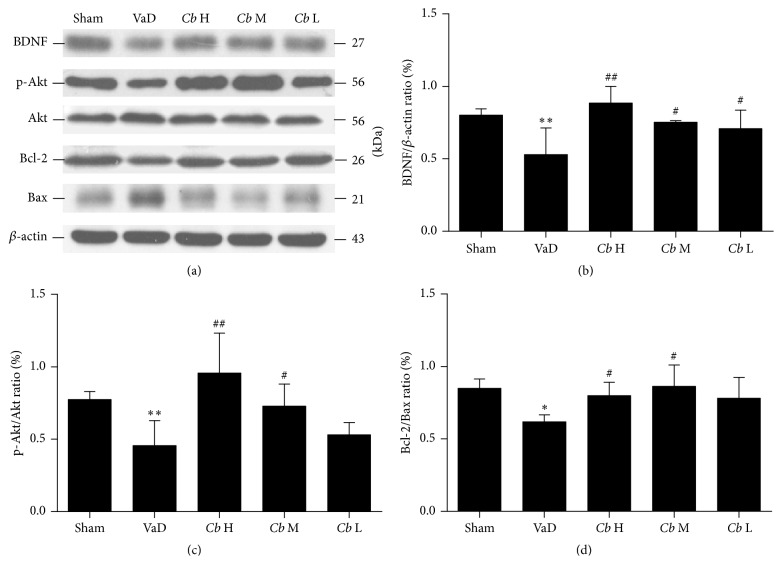
Western blot analysis. (a) Western blot of the expression levels of BDNF, p-Akt, Akt, Bcl-2, and Bax in mouse hippocampus. (b) A quantitative analysis of the protein levels of BDNF from each group normalized to the loading control *β*-actin (c). (d) Bar graphs showing the protein level ratios of p-Akt/Akt and Bcl-2/Bax from each group; Sham, sham-operated group; VaD, VaD model group; *Cb* H,* C. butyricum-* (1 × 10^8^ CFU-) treated group; *Cb* M,* C. butyricum-* (1 × 10^7^ CFU-) treated group; and *Cb* L,* C. butyricum-* (1 × 10^6^ CFU-) treated group. Error bars indicate SD; *n* = 8 for each group. ^*∗*^ *P* < 0.01 and ^*∗∗*^ *P* < 0.01 versus sham group; ^#^ *P* < 0.05 and ^##^ *P* < 0.01 versus VaD group.

Using Western blot analysis, the protein level of BDNF in the VaD group was significantly decreased compared with the sham group (*P* < 0.01, Figures 5(a) and 5(b)), whereas that of the* C. butyricum*-treated mice was significantly increased compared with the mice with VaD (1 × 10^6^ and 1 × 10^7^ CFU: *P* < 0.05; 1 × 10^8^ CFU: *P* < 0.01, Figures 5(a) and 5(b)). The ratio of p-Akt/Akt was significantly decreased in the VaD group in comparison with the sham (*P* < 0.01, Figures 5(a) and 5(c)). The ratio of p-Akt/Akt in the* C. butyricum-* (1 × 10^7^ CFU: *P* < 0.05; 1 × 10^8^ CFU: *P* < 0.01-) treated mice was significantly increased compared with the VaD group (Figures 5(a) and 5(c)), suggesting that* C. butyricum* could activate Akt. The ratio of Bcl-2/Bax (antiapoptotic/proapoptotic) was significantly reduced in the VaD group compared with the sham (*P* < 0.05, Figures 5(a) and 5(d)). However, the ratio in the* C. butyricum-* (1 × 10^7^ and 1 × 10^8^ CFU-) treated mice was remarkably increased compared with the VaD group (*P* < 0.05, Figures 5(a) and 5(d)), whereas there was no significant difference between* C. butyricum-* (1 × 10^6^ CFU-) treated mice and the VaD group (*P* > 0.05, Figures 5(a) and 5(d)).

**Figure 6:**
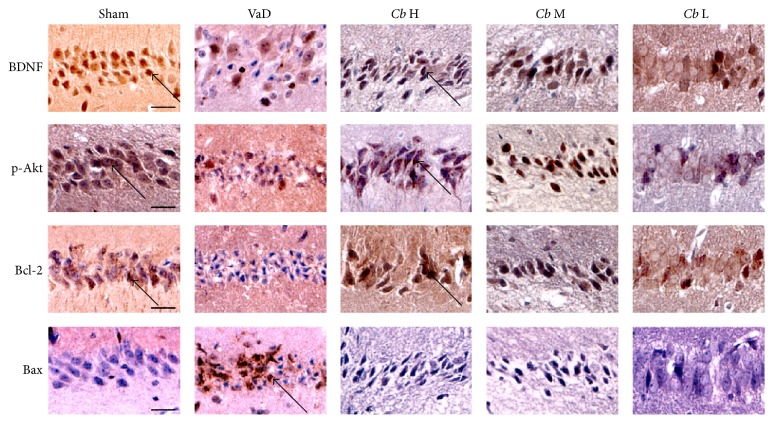
Immunohistochemical staining. Representative images of the immunohistochemical staining of BDNF, p-Akt, Bcl-2, and Bax in area CA1 of the hippocampus. Cells with a brown-stained cytoplasm are considered positive. Sham, sham-operated group; VaD, VaD model group; *Cb* H,* C. butyricum-* (1 × 10^8^ CFU-) treated group; *Cb* M,* C. butyricum-* (1 × 10^7^ CFU-) treated group; and *Cb* L,* C. butyricum-* (1 × 10^6^ CFU-) treated group. Magnification: 400x. Scale bar = 20 *μ*m.

Using immunohistochemistry, as shown in Figure 6, few Bax-positive cells could be found in the sham group, whereas most cells were positive for BDNF, p-Akt, and Bcl-2. In the VaD group, there were a large number of Bax-positive cells, while the staining for BDNF, p-Akt, and Bcl-2 was reduced compared with the sham group in the hippocampal CA1 region. After the* C. butyricum* treatment, the number of the Bax-positive cells was significantly decreased, whereas the number of cells positive for BDNF, p-Akt, and Bcl-2 was significantly increased in comparison with the VaD group.

### 3.4. *C. butyricum* Increased the Diversity of Intestinal Bacteria

**Figure 7:**
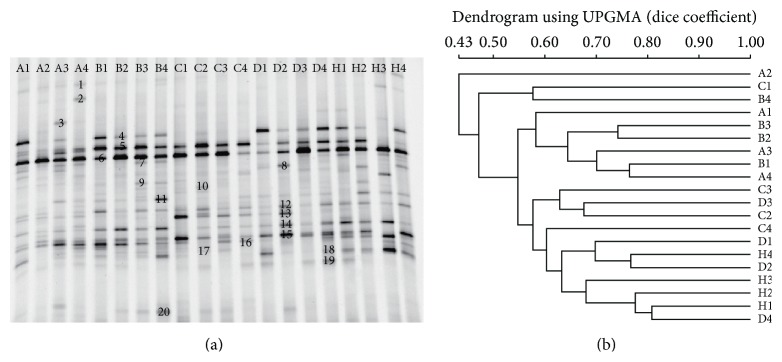
PCR-DGGE analysis of the predominant fecal microbiota in mice. (a) PCR-DGGE fingerprints used to analyze the fecal microbiota of the samples from the VaD model group (A), sham-operated group (H), and* C. butyricum-* (B, 1 × 10^6^ CFU-; C, 1 × 10^7^ CFU-; and D, 1 × 10^8^ CFU-) treated groups. Each lane represents one subject that was randomly selected from each group. The bands marked in the DGGE gel were identified by cloning and sequencing to facilitate the interpretation of the figure. Bands: 1:* Bacteroides* sp.; 2:* Hespellia* sp.; 3:* Clostridium* XVIII sp.; 4: TM7 genera incertae sedis; 5: TM7 genera incertae sedis; 6:* Anaerostipes* sp.; 7:* Barnesiella* sp.; 8:* Barnesiella* sp.; 9:* Roseburia* sp.; 10:* Acinetobacter* sp.; 11: unclassified* Helicobacteraceae* sp.; 12:* Streptobacillus* sp.; 13:* Barnesiella* sp.; 14:* Clostridium* XIVa sp.; 15:* Alistipes* sp.; 16: unclassified* Lachnospiraceae* sp.; 17:* Lachnospiraceae* incertae sedis* Alistipes* sp.; 18:* Anaerostipes* sp.; 19: unclassified* Porphyromonadaceae* sp.; and 20:* Butyricimonas* sp. (b)* Dendrogram* of the DGGE profiles shown in (a).

As shown in Figure 7, the PCR-DGGE profiles revealed that the overall structure and diversity of the predominant fecal bacteria changed drastically after the* C. butyricum* treatment. There were fewer bands in the VaD group than in the sham group, indicating a dramatically decreased level of bacterial diversity. However, the* C. butyricum* treatment clearly increased the bacterial diversity, particularly in* C. butyricum-* (1 × 10^8^ CFU-) treated mice. The cluster analysis of the DGGE profiles, which was based on the similarity indices, showed that samples from the sham mice and the* C. butyricum-* (1 × 10^7^ CFU and 1 × 10^8^ CFU-) treated mice clustered together in one branch, whereas the samples from the mice with VaD and the* C. butyricum-* (1 × 10^6^ CFU-) treated mice clustered in a separate branch, although one of the* C. butyricum-* (1 × 10^7^ CFU-) treated sample clusters was grouped with the VaD group. These results demonstrated that there were dramatic changes in the richness and diversity of the predominant fecal bacteria in the* C. butyricum-* (1 × 10^7^ CFU and 1 × 10^8^ CFU-) treated mice, which indicated the restoration of fecal microbiota after* C. butyricum* treatment. The bands excised from the DGGE gel represented the predominant fecal microbiota in mice.

### 3.5. *C. butyricum* Increased the Level of Butyrate in the Feces

**Figure 8:**
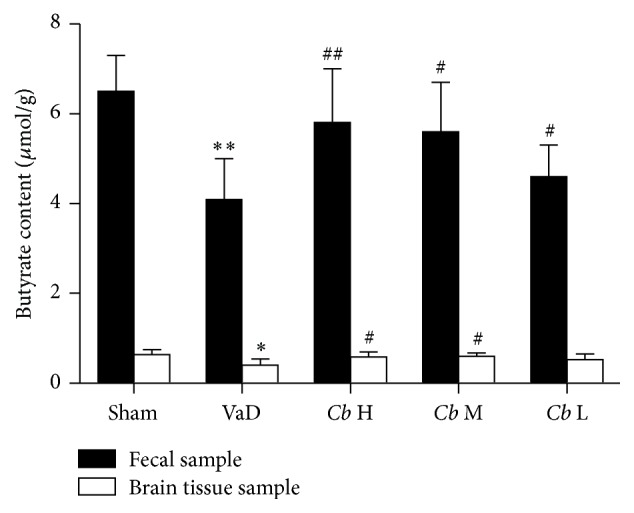
Absolute concentration of butyrate (*μ*mol g^−1^ wet weight). Sham, sham-operated group; VaD, VaD model group; *Cb* H,* C. butyricum-* (1 × 10^8^ CFU-) treated group; *Cb* M,* C. butyricum-* (1 × 10^7^ CFU-) treated group; and *Cb* L,* C. butyricum-* (1 × 10^6^ CFU-) treated group. Error bars indicate SD; *n* = 8 for each group. ^*∗*^ *P* < 0.05 versus sham group, ^*∗∗*^ *P* < 0.01 versus sham group, and ^#^ *P* < 0.05 and ^##^ *P* < 0.01 versus VaD group.

The absolute concentrations of the fecal butyrate significantly decreased in the mice with VaD compared with the sham mice (*P* < 0.01, Figure 8), whereas* C. butyricum* treatment (1 × 10^6^ CFU and 1 × 10^7^ CFU: *P* < 0.05; 1 × 10^8^ CFU: *P* < 0.01, Figure 8) resulted in a significant increase in the fecal butyrate level.

### 3.6. *C. butyricum* Increased the Level of Butyrate in the Brain

The concentration of butyrate in the brains of mice with VaD was significantly decreased compared with the sham mice (*P* < 0.05, Figure 8). However, the concentration of butyrate in the brains of* C. butyricum-*treated mice (1 × 10^7^ CFU and 1 × 10^8^ CFU: *P* < 0.05, Figure 8) was remarkably increased, whereas there was no significant difference between* C. butyricum-* (1 × 10^6^ CFU-) treated mice and the VaD group (*P* > 0.05, Figure 8). These results indicate that higher quantitative* C. butyricum* could elevate butyrate levels in the brains of mice with VaD.

## 4. Discussion

Probiotics are dietary supplements that exert beneficial effects under various clinical conditions including astriction, dysbacteriosis, and antimicrobial-associated diarrhea in humans and animals [18, 22]. However, the widespread use of probiotics for conditions outside of the gastrointestinal system is lacking, due to little mechanistic insight [21]. This study demonstrates, for the first time, that VaD is associated with gut microbiota. In this study, we evaluated the neuroprotective effects of the probiotic* C. butyricum* and the production of SCFAs, namely, butyrate, which has been widely used to modulate intestinal dysbacteriosis against rUCCAO-induced VaD in mice. The beneficial metabolic neuroprotective effects of* C. butyricum* occurred via changes in the microbiota population resident in the intestinal tract of* C. butyricum*-treated mice. We confirmed that* C. butyricum* treatment could ameliorate both the cognitive impairment and histopathologic changes of area CA1 of the hippocampus in mice with VaD. The altered gut microbiotas, especially those that have the ability to influence the probiotic-gut microbiota-butyrate-brain axis, stimulated the production of butyrate that in turn elevated butyrate levels in the brain to protect against VaD in mice.

The improvement of cognitive function is a desirable target for therapies against VaD. The rUCCAO-inducted VaD mouse model is characterized by damage to the hippocampus that is caused by cerebral hypoperfusion and results in cognitive deficits [27]. The role of probiotics in reducing depressive and anxiety-like conditions is a region of active research [7, 13]. Our results showed that* C. butyricum* treatment could improve the cognitive function and performance of mice with VaD in the Morris water maze, suggesting that* C. butyricum* is involved in alleviating cognitive deficits.

Apoptosis is coincident with the development of ischemic VaD, and protection against apoptosis plays an important role in the recovery from VaD [34]. In this study, we observed a reduction in the histopathologic changes and the degree of apoptosis in* C. butyricum*-treated mice using electron microscopy with HE and TUNEL staining. These findings could partially account for the neuroprotective effects observed in* C. butyricum*-treated mice. To gain mechanistic insight, we investigated the expression patterns of BDNF-PI3K/Akt pathway-related proteins (BDNF, p-Akt, Akt, Bcl-2, and Bax) in the brain. The PI3K/Akt signaling pathway, which is a central mediator that regulates neuronal growth, survival, and metabolism [35], has been widely reported to participate in the protection against VaD. BDNF, a member of the neurotrophin family, exerts a protective effect against ischemic brain injury via antiexcitatory amino acids, the inhibition of inflammatory reactions, and decreased apoptosis [36, 37]. Recent evidence has indicated that the reduction of BDNF can aggravate anxiety-like behavior in mice [38]. In this study, we found that the expression of BDNF in mice with VaD was significantly decreased compared with the sham group, whereas* C. butyricum* treatment increased BDNF levels compared with the VaD group, suggesting that higher BDNF levels are related to the reduction in behavioral deficits and apoptosis in mice with VaD. In addition, BDNF can activate various intracellular signaling cascades including the PI3K/Akt signal pathway [39]. Our study indicates that* C. butyricum* treatment could enhance the levels of p-Akt and activate Akt in mice with VaD. The activation of Akt suppresses apoptosis and promotes cell survival by regulating downstream targets such as Bcl-2 and Bax [40]. Bcl-2 is an antiapoptotic factor that is important for cell survival while Bax promotes apoptosis [41]. Our results show that* C. butyricum* treatment significantly increased the levels of Bcl-2 and decreased the levels of Bax in mice with VaD. Taken together, these results indicate that the beneficial effects of* C. butyricum* were attributable to the reduction in neuronal apoptosis and that the BDNF-PI3K/Akt pathway could be involved in this antiapoptotic effect. It is possible that* C. butyricum* indirectly ameliorated the histopathologic changes and apoptosis in mice with VaD.

Probiotics mainly present their beneficial effects via the modulation of gut microbiota [7]. Some psychiatric diseases, such as autism, depression, anxiety, and stress, had been reported to induce a varying degree of gut microbiota alterations in animals and even humans [7, 13, 14]. In this study, we observed a significant increase in gut microbiota diversity in the* C. butyricum*-treated mice. A cluster analysis of DGGE profiles, which was based on the similarity indices, showed that the samples from the sham group and the* C. butyricum-*treated mice (1 × 10^7^ or 1 × 10^8^ CFU) clustered together in one branch, whereas the other samples from the mice with VaD and the 1 × 10^6^ CFU* C. butyricum*-treated mice clustered on a different branch, although one of the 1 × 10^7^ CFU* C. butyricum* sample clusters was grouped with the VaD mouse model. However, it is plausible that other bacterial populations were significantly changed upon* C. butyricum* administration, and further metagenomic studies will provide a comprehensive understanding of the microbiome changes elicited by* C. butyricum*. These results demonstrated that there were dramatic changes in the richness and diversity of the predominant intestinal bacteria in the* C. butyricum-*treated mice (1 × 10^7^ or 1 × 10^8^ CFU), which indicated the restoration of gut microbiota after* C. butyricum* treatment.

In order to connect* C. butyricum*, gut microbiota activity, and VaD, we investigated the microbiota composition as well as its major products of fermentation, the SCFAs. SCFAs and fecal butyrate in mice with VaD were significantly decreased. A significant increase of fecal butyrate in* C. butyricum*-treated mice was observed. SCFAs, such as acetate, propionate, and butyrate, are known bioactive metabolites of fermentation of the bacterial population residing in the gastrointestinal tract. SCFAs have diverse beneficial metabolic effects [42]. The levels of SCFAs in the gastrointestinal tract are influenced by the microbial composition [23]. As with the occurrence of intestinal dysbiosis, the level of fecal SCFAs in the intestinal tract may change or decrease accordingly. Probiotics, such as* C. butyricum, Lactobacilli*, and* Bifidobacterium*, produce abundant SCFAs from the fermentation of fibers. Changes in SCFAs have been associated with gut microbiota modulation in other studies [24, 43]. Moreover, fecal butyrate levels were significantly increased in* C. butyricum*-treated mice. Our previous experimental results have indicated that administration of sodium butyrate significantly attenuates oxidative stress, inflammation, and neuronal apoptosis and improves neurological deficits in mice with a cerebral I/R injury induced by bilateral common carotid artery occlusion (BCCAO). Altogether, these observations suggest that gut-derived butyrate plays an important role in the treatment of VaD. We hypothesize that an increased colonization of butyrate-producing bacteria occurs in mice administered* C. butyricum*. However, extensive metagenomic analyses are needed to determine the type of bacteria responsible for the butyrate production.

Interestingly, in this study, after the* C. butyricum* treatments, there was significant rise of butyrate content in the feces and in the brains. A rise of butyrate content in the brain may be another mechanism of* C. butyricum* against VaD in mice. However, how does butyrate perform neuroprotective effects? We hypothesized that the rise of butyrate in the brains is due to the changes in the microbiota composition, and the butyrate travels from the intestinal tract to the brains to exert neuroprotective effects. Butyrate can cross the blood-brain barrier to modulate CNS functions including brain development and behavior [17, 43]. Butyrate might play an important role in the CNS [24, 44]. A recent study suggests that butyrate, as an inhibitor of histone deacetylase (HDAC), improves spatial learning and memory ability and can provide antiapoptotic and neuroprotective effects against ischemic stroke [45]. Little is known regarding the interactions between gut microbiota and their ability to elicit neuroprotective effects [23, 46]. Our experimental results showed that* C. butyricum* treatments could modulate the gut microbiota, and a relation was observed between improvement of brain function and changes in the microbiota composition. However, a further study to investigate this potential mechanism is necessary to be carried out.

## 5. Conclusion

In summary, this study provides data to support the use of* C. butyricum* as a safe and economical therapeutic option against VaD, especially its ability to influence the gut microbiota-butyrate-brain axis, to prevent and treat VaD in mice. The possibility that dietary* C. butyricum* can regulate gut microbiota and lead to changes in the fecal butyrate content that raise butyrate in the brain will further incite exploration aimed at understanding the mechanisms underlying VaD recovery. These results open new avenues for viable therapeutic options against VaD by gut microbiota modulation. Therefore, the administration of live* C. butyricum* may become an adjuvant therapy for VaD patients.
